# Impaired auditory sampling in dyslexia: further evidence from combined fMRI and EEG

**DOI:** 10.3389/fnhum.2013.00454

**Published:** 2013-08-09

**Authors:** Katia Lehongre, Benjamin Morillon, Anne-Lise Giraud, Franck Ramus

**Affiliations:** ^1^Research Center of the Brain and Spine Institute (CRICM), Hôpital de la Pitié-SalpêtrièreParis, France; ^2^Department of Psychiatry, Columbia University Medical CenterNew York, NY, USA; ^3^Department of Neuroscience, University of GenevaGeneva, Switzerland; ^4^Laboratoire de Sciences Cognitives et Psycholinguistique, Ecole Normale Supérieure, EHESS, CNRSParis, France

**Keywords:** dyslexia, auditory sampling, phonemic processing, gamma oscillation, theta oscillation, delta oscillation, EEG-fMRI

## Abstract

The aim of the present study was to explore auditory cortical oscillation properties in developmental dyslexia. We recorded cortical activity in 17 dyslexic participants and 15 matched controls using simultaneous EEG and fMRI during passive viewing of an audiovisual movie. We compared the distribution of brain oscillations in the delta, theta and gamma ranges over left and right auditory cortices. In controls, our results are consistent with the hypothesis that there is a dominance of gamma oscillations in the left hemisphere and a dominance of delta-theta oscillations in the right hemisphere. In dyslexics, we did not find such an interaction, but similar oscillations in both hemispheres. Thus, our results confirm that the primary cortical disruption in dyslexia lies in a lack of hemispheric specialization for gamma oscillations, which might disrupt the representation of or the access to phonemic units.

## Introduction

Developmental dyslexia is a specific difficulty in the acquisition of reading skills that is not accounted for by mental age, visual acuity deficit or inadequate schooling (WHO, 2011). It is thought to affect between 3 and 7% of the population (Lindgren et al., 1985), although estimates vary widely depending on cut-off criteria on reading performance scales.

While there remains a great diversity of theoretical outlooks on dyslexia (Ramus and Ahissar, 2012), it is now widely agreed that the majority of dyslexic individuals share difficulties in one or several aspects of phonological processing, including paying attention to and mentally manipulating speech sounds (phonological awareness), storing phonological material for a few seconds (verbal short-term memory), and rapidly retrieving long-term phonological representations (Wagner and Torgesen, 1987; Vellutino et al., 2004). Debates persist on whether this phonological deficit (i) follows from an underlying primary auditory processing deficit (Tallal, 1980; Goswami et al., 2011), (ii) arises from a degradation of phonological representations or in difficulties accessing them (Ramus and Szenkovits, 2008), and (iii) is sufficient by itself to account for learning disability or merely a risk factor (Pennington, 2006).

At the anatomo-functional level, while the issue remains debated (Richlan et al., 2011), the vast majority of findings from functional and structural brain imaging and post-mortem dissection converge on a disruption of left perisylvian cortical networks, which are involved in speech processing and recruited for reading acquisition (Galaburda et al., 1985; Eckert, 2004; Richardson and Price, 2009; Linkersdorfer et al., 2012). Genetic variations associated with dyslexia further point to structural and functional variations in the perisylvian language network (Darki et al., 2012; Pinel et al., 2012; Giraud and Ramus, 2013).

A new theoretical framework for the cortical organization of speech processing sheds a new light on the neural basis of developmental dyslexia. According to “asymmetric sampling in time” (AST) theory (Poeppel, 2003), left and right auditory cortices show cortical oscillations at different preferred rates: low gamma (25–45 Hz) in the left hemisphere and delta-theta (1–7 Hz) in the right. Gamma and theta oscillations are assumed to play a role in the segmentation of the sound stream into units of the corresponding sizes, which may be optimal for the analysis of phonemes by the left auditory cortex and syllables and prosodic cues by the right one (around 25 and 200 ms, respectively). While this hypothesis has received empirical support from neuroimaging and neurophysiological studies (Giraud et al., 2007; Abrams et al., 2008; Telkemeyer et al., 2009; Morillon et al., 2010), its implications for developmental dyslexia are not so clear. On the one hand, Giraud and Poeppel (2012) have hypothesized that dyslexic individuals might not show the typical left-hemisphere specialization for the gamma rate, which would explain their specific difficulties with the processing of phonemic units. On the other hand, Goswami (2011) has hypothesized that dyslexic individuals might rather show atypical cortical oscillations in the theta (4–7 Hz) or in the delta (1–4 Hz) frequency band, thereby leading to processing deficits at the syllable and more generally at the prosodic level, which are also proposed to be causally linked to reading disability (Goswami et al., 2010).

In a previous study, we used magnetoencephalography (MEG) to measure auditory steady-state responses (ASSRs) to an amplitude-modulated white noise in dyslexic and control individuals. We found the expected left dominance of ASSRs in the *planum temporale* in the gamma range (25–35 Hz) in controls, but not in dyslexic participants who displayed no or a reversed asymmetry (Lehongre et al., 2011). These results, that are supported by another study that also measured ASSRs [using electroencephalography (EEG)] and found group differences at 20 Hz in the left hemisphere (Poelmans et al., 2012), were consistent with the hypothesis of a left-hemisphere-based gamma oscillation disruption, but did not explicitly test the delta/theta alteration hypothezis. In the delta/theta range, one study found no group difference at 4 Hz using EEG-ASSR (Poelmans et al., 2012), and another one exploring MEG-ASSRs at 2, 4, 10 and 20 Hz found a right-hemisphere dominance at 2 Hz in controls but not in dyslexic participants and no further group difference at any other frequency (Hamalainen et al., 2012). Using more naturalistic speech stimuli, an EEG study found that poor readers, in contrast to good readers, did not present the natural dominance of the right hemisphere to follow the speech envelope. They presented a more bilateral pattern, but this was the case only when speech was compressed to 50% of its original duration (Abrams et al., 2009). Finally, anomalies in the alpha rhythm have also been found in children with dyslexia or with language impairments (Heim et al., 2011; Babiloni et al., 2012; Han et al., 2012).

In the context of these conflicting results, the goals of the present study were twofold: Firstly, to replicate and extend the results of our previous study, using more naturalistic stimuli and an entirely different methodology; secondly, to directly compare the predictions of the two main theories by testing cortical oscillations in the gamma, theta and delta ranges in auditory cortices. For this purpose, rather than using a simple amplitude-modulated noise, we exposed participants to an audiovisual documentary movie featuring more ecological conversational speech, thereby including the whole range of amplitude modulations relevant to language.

We used simultaneous EEG and functional magnetic resonance imaging (fMRI) to measure the activity of the auditory cortex at both high temporal (millisecond) and spatial (millimeter) resolution. Changes in Blood-oxygen-level-dependent (BOLD) are associated with changes in the spectral profile of neuronal activity (Rosa et al., 2010b), and EEG and fMRI do not capture exactly the same signals. Only a small fraction of the BOLD signal reflects synaptic activity involving pyramidal cells (Lee et al., 2010; Logothetis, 2010), which we hypothesize here to work as a sampling device. By combining delta, theta and low-gamma power variations and BOLD activity we expect to capture the part of the BOLD activity that is explained out by circuits involving pyramidal cells, which generate these rhythms. Furthermore, simultaneous EEG/fMRI recordings are well suited to study oscillations in continuous recordings with uncontrolled states as it has been demonstrated in several studies [see for review: (Herrmann and Debener, 2008; Rosa et al., 2010a; Laufs, 2012)].

## Materials and methods

### Participants

Thirty-two normal-hearing, French–native-speakers participated in a simultaneous EEG and fMRI experiment (with approval of the local ethics committee; biomedical protocol C08–39). Seventeen participants reported a history of reading disability, scored at or below the expected level for ninth graders in a standardized reading test, and at least 90 in non-verbal IQ. The remaining 15 participants were normal readers matching dyslexic participants with respect to age, gender, handedness, and non-verbal IQ, but scoring above the ninth grade reading level. Demographic and psychometric data, as well as the results of a larger battery (Soroli et al., 2010) of literacy and phonological tests are reported in Table 1.

**Table 1:** **Summary of behavioral tests**.

	**Controls (15 subjects)**			**Dyslexics (17 subjects)**		
	***n***	**Mean**	**sd**	***n***	**Mean**	**sd**
Males	7			9		
Right handed	10			12		
Age	15	24.09	3.54	17	23.79	4.04
Non-verbal IQ	15	112.07	13.43	17	111.35	10.73
EVIP Vocabulary	15	123.07	2.94	15	120.67	5.26
Reading fluency (nb of correct words/min)^***^	15	196.16	36.98	17	113.53	24.70
Spelling (%)^**^	15	94.22	4.67	12	81.25	11.81
Orthographic choice accuracy (%)	14	95.07	5.47	15	89.60	9.82
RAN of objects (sec)^**^	15	61.80	10.23	17	78.37	17.85
RAN of digits (sec)^***^	15	31.87	6.56	17	44.78	12.34
RAN of colors (sec)^**^	15	54.33	9.15	17	71.21	18.80
Spoonerisms accuracy^**^	15	0.75	0.10	15	0.50	0.26
Digit span (%)^***^	15	10.73	1.58	17	7.35	2.85

*As indicated by the number of participants, some values were missing for some tests*.

*Sec: seconds; Stars indicate significant differences between controls and dyslexics (independent t-test*,

** *for p < 0.01*,

*** *for p < 0.001, uncorrected)*.

### Behavioral test battery

Non-verbal intelligence was assessed in all participants using Raven's matrices (Raven et al., 1998). Their receptive vocabulary was assessed with the EVIP test (Dunn et al., 1993), a French adaptation of the Peabody picture vocabulary scale. They were included on the basis of performance on the Alouette test (Lefavrais, 1967), a meaningless text that assesses both reading accuracy and speed, yielding a composite measure of reading fluency. Orthographic skills were assessed using a computerized orthographic choice task, and a spelling-to-dictation test. Phonological tests: we used the WAIS digit span as a measure of verbal working memory (Wechsler, 2000). Phonological awareness was assessed using a computerized spoonerism task, in which participants heard pairs of words, and had to produce them swapping the initial phonemes. Finally, rapid automatized naming was assessed using the object and digit sheets from the PhAB (Frederickson et al., 1997) and two custom-made color sheets modeled on the object sheets, with five different colors repeated 10 times each, one replacing each object. The dependent variable is the total time taken to name all items on each sheet, irrespective of errors.

### MRI acquisition

Subjects were either asked to rest with closed eyes or to pay attention to an audiovisual movie (Morillon et al., 2010). The movie, a scientific documentary on an ecological topic, included three speakers (2 men). Data were acquired in three sessions with session 1: 10 min of rest; sessions 2 and 3: 10 min of movie followed by 11 min of rest. The scanner produced a constant auditory signal during data acquisition presumed not to interfere with our analyzes, given that its modulation frequencies are outside our frequencies of interest (See supplemental data p. 1, Morillon et al., 2010).

The three sessions yielded 1560 echoplanar fMRI image volumes (Tim-Trio; Siemens, 40 transverse slices, voxel size = 3 × 3 × 3 mm; repetition time = 2 s; echo time = 50 ms; field of view = 192). A 7-min anatomical T1-weighted magnetization-prepared rapid acquisition gradient echo sequence (176 slices, field of view = 256, voxel size = 1 × 1 × 1 mm) was acquired at the end of scanning.

### MRI preprocessing

We used statistical parametric mapping (SPM8; Wellcome Department of Imaging Neuroscience, UK; www.fil.ion.ucl.ac.uk) for fMRI standard preprocessing, which first involved realignment of each subject's functional images and coregistration with structural images. Structural images were segmented, spatially normalized to Montreal Neurological Institute stereotactic space to 3 × 3 × 3 mm and finally spatially smoothed with a 10-mm full-width half-maximum isotropic Gaussian kernel to compensate for residual variability after spatial normalization.

### EEG acquisition

Continuous EEG data were recorded at 5 kHz from 62 scalp sites (Easycap electrode cap) using MR compatible amplifiers (BrainAmp MR and Brain Vision Recorder software; Brainproducts). Two additional electrodes (electro-oculograph, EOG and electrocardiograph, ECG) were placed under the right eye and on the collarbone. FCz was set as reference for the acquisition of all electrodes. Impedances were kept under 10 kΩ and EEG was time-locked with the scanner clock, which helps to have a very reproducible, more easily removed MRI artifact, thus resulting in higher EEG quality in the gamma band (Mandelkow et al., 2006).

### EEG preprocessing

Gradient and pulse artifacts were first detected and marked using in-house software (wiki.cenir.org/doku.php/datahandler). Detection was achieved by correlating the data with automatically (for gradient) or manually (for pulse) defined templates. We used the raw signal of channel FC2 for gradient artifacts, and the raw ECG signal filtered between 0.5 and 10 Hz for pulse artifacts. In a second step, artifacts were corrected using two software: FASST v111017 (www.montefiore.ulg.ac.be/ phillips/FASST.html) for gradient artifacts and EEGlab v.9 (sccn.ucsd.edu/eeglab) for pulse artifacts. In both cases we used the principal component analyses methods implemented in the software. Blinks and vertical eye movements were corrected using independent component analyses as described in EEGlab's tutorial. Data were subsequently down-sampled to 250 Hz and re-referenced to a common average reference. The original reference electrode was recalculated as FCz, generating a total of 63 cortical electrodes.

For each subject, periods with head movement artifacts were detected by visual inspection, for rejection purposes as described below. As our interest was in auditory activity, we focused the analyses described below on the temporal channels that best captured the activity of our ROIs, i.e., where we observed the highest correlation between EEG and BOLD: T7, T8, FT7, FT8, TP7, TP8, TP9, and TP10.

### Analyzes

Analyzes are reported for the movie condition, as our interest was to explore brain oscillation properties during natural speech processing. Analyzes were also conducted on rest sessions but did not yield any significant result, in particular no group effect.

### Time-frequency analyzis

For each movie session, we performed time-frequency (TF) analyzes with a Morlet wavelets approach using Fieldtrip (Oostenveld et al., 2011) at each channel for frequencies from 1 to 48 Hz bands, with a frequency step of 1 Hz and a time step of 0.1 s. For each frequency and channel, the previously detected periods of movement were rejected by replacing values by NaNs (Not A Number). The power time courses were converted to Z-scores, which, when larger than 4, were replaced by NaNs to remove residual artifacts. The transformed signal was then averaged over the eight temporal channels, Z-transformed a second time and averaged across frequency bands of interest: 1–3 Hz (delta), 4–7 Hz (theta) and 25–35 Hz (gamma). Finally NaNs were replaced by zeros. We removed (on average across all subjects) around 40 s of signal per movie condition (mean +/− s.e.m: controls: 36 +/− 8 s; dyslexics: 45 +/− 18 s; independent *t*-test: *t* = −0.44, *p* = 0.65).

### Regions of interest (ROI)

**Figure 1:**
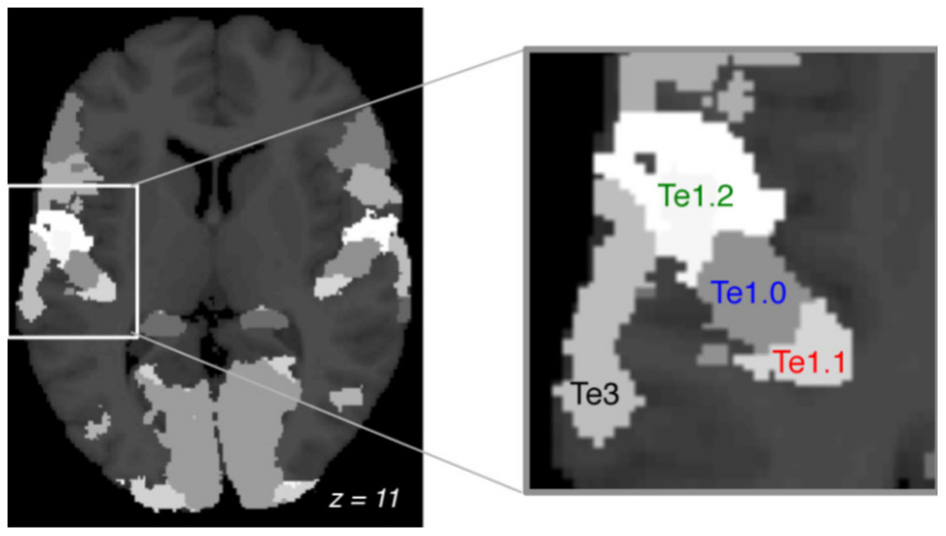
**Regions of interest.** Cortical cytoarchitectonic areas defined by the anatomy toolbox (axial plane, MNI: z = +11). Focus on the left auditory cortex and the four ROIs: Te1.1, Te1.0, Te1.2, Te3. Te1 is Heschl's gyrus and Te3 is the *planum temporale*.

In accordance with AST theory we focused our analysis on the auditory cortex. We used three regions of interest (ROI) within Heschl's gyrus (Te1.0, Te1.1, and Te1.2), and the *planum temporale*: Te3 (Figure 1), all defined from cytoarchitectonic criteria using the SPM anatomy toolbox v.1.6. BOLD time courses were averaged over voxels using MarsBar (Brett et al., 2002).

### Correlation between bold time course of ROIs and EEG power spectrum

For each ROI and frequency band (delta, theta and gamma), we built a design matrix to perform a partial correlation between the BOLD time course of the ROI and the EEG power. The EEG time courses (averaged across all eight electrodes) of each frequency band were convolved with the hemodynamic response function (HRF) and downsampled to 0.5 Hz, i.e., the fMRI sampling rate. This analyzis was meant to capture the degree to which each ROI oscillates in each frequency band. A positive correlation indicates that the EEG power fluctuations in any given frequency band are reflected in the modulations of local synaptic activity as detected with fMRI (Laufs et al., 2003). Such correlations allow us to exploit the spatially detailed BOLD effect to precisely localize widespread EEG effects, resulting in a finer localization of oscillatory activity than with EEG only (Rosa et al., 2010a). We averaged over the whole set of temporal electrodes (left and right hemisphere) to avoid hemispheric biases in the EEG. Using this approach, asymmetries arise from specific correlations with the BOLD effect (Giraud et al., 2007). Our statistical model also included the motion parameters, their derivatives, the averaged signal of three brain compartments (white-matter gray-matter and CSF), and of all out-of-brain voxels, as nuisance covariates, as well as a regressor modeling the sessions. For normalization purposes the entire matrix was Z-transformed in time.

### Group level statistics

A repeated-measures ANOVA (with Greenhouse-Geisser corrections when appropriate) was performed with SPSS (IBM Corp. Released 2012. IBM SPSS Statistics for Windows, Version 21.0. Armonk, NY), with the Fisher transformed partial correlation coefficients between EEG power and BOLD time courses as dependent variables, the 4 areas, 3 frequency bands and 2 hemispheres as within-subject factors, and group, sex and handedness as between-subject factors. All factors were entered as main effects in the model, as well as the 2nd, 3rd, and 4th order interactions of the 3 within-subject factors and group. Effect sizes were estimated for some measures of within or between group differences with Cohen's formula: *d* = (*M*_1_–M_2_)/SD_pooled_, with M_1_ = mean of group 1, M_2_ = mean of group 2, SD_pooled_ = √[(SD_1_^2^ + SD_2_^2^)/2], SD_1_ = standard deviation of group 1, SD_2_ = standard deviation of group 2. All other analyses were done using Matlab (version 2011/2012) (The MathWorks Inc., Natick, Massachusetts, USA).

## Results

The repeated-measures ANOVA yielded significant main effects of area and hemisphere, and a group × frequency × hemisphere interaction.

**Figure 2:**
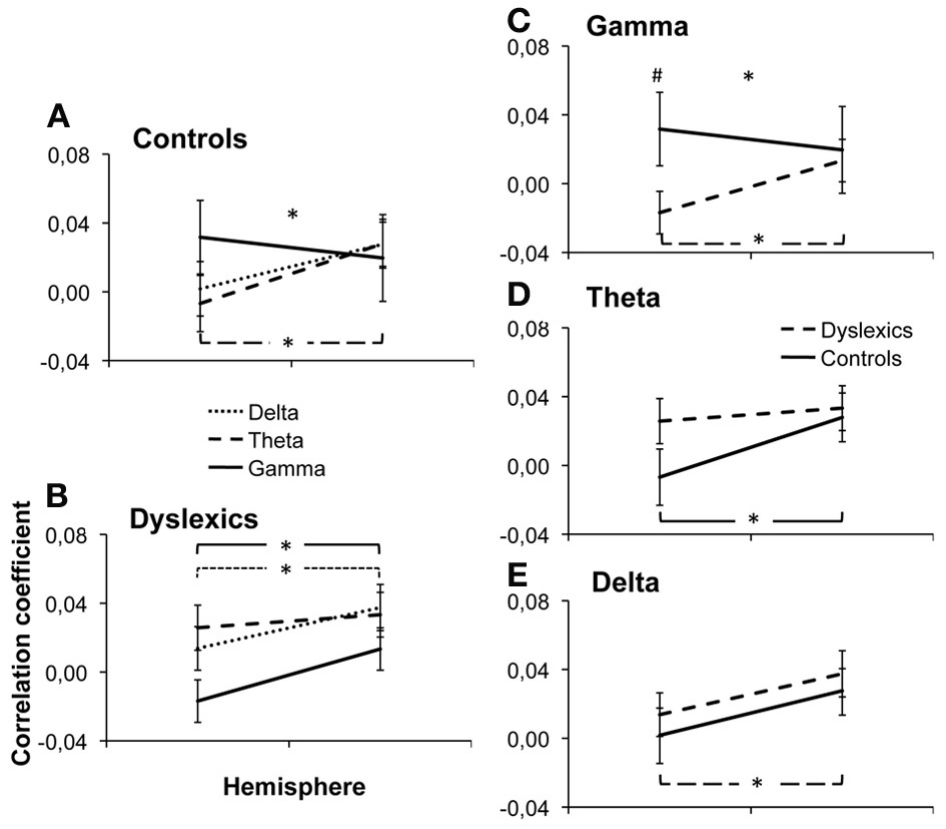
**Group × Frequency × Hemisphere interaction.** Individual beta values were averaged over ROIs and plotted along the different dimensions of the interaction: Frequency × Hemisphere interaction in controls **(A)** and dyslexics **(B)** and Frequency × Group interaction in the gamma **(C)**, delta **(D)** and theta **(E)** bands. Error bars represent the s.e.m. ^*^indicates a significant interaction or difference with *p* < 0.05. ^#^indicates a marginally significant difference, *p* = 0.066.

Across groups, frequencies and hemispheres, there was a main effect of area [*F*_(2.2, 26.8)_ = 7.45, *p* = 0.001]. Correlations between EEG and fMRI were overall strongest in area Te1.2 and weakest in area Te1.1. This suggests that different regions of the auditory cortex show differential strength of responses in the frequency bands of interest, however, the specific correlation pattern observed here does not suggest any obvious interpretation. We also observed a main effect of hemisphere, with overall higher correlations in the right than in the left hemisphere [*F*_(1, 28)_ = 8.28, *p* = 0.008], an effect that seems to be carried by the delta and theta bands [*F*_(1,28)_ = 10.15, *p* = 0.004 and F_1,28_ = 7.04, *p* = 0.013 respectively, see Figure 2].

Most interestingly, we observed a group × hemisphere × frequency band interaction [*F*_(1.90, 27.10)_ = 6.56, *p* = 0.003, Figure 2, Table 2. Controls presented a frequency × hemisphere interaction [*F*_(1.76, 11.24)_ = 5.21, *p* = 0.017, Figure 2A], with right dominance for theta [*F*_(1, 12)_ = 8.71, *p* = 0.012]. The delta and gamma bands showed no significant asymmetry [*F*_(1, 12)_ = 3.10, *p* = 0.104; F_1, 12_ = 0.51, *p* = 0.487, respectively]. Conversely, dyslexics showed the same correlation patterns across frequencies [frequency × hemisphere: *F*_(1.93, 13.07)_ = 1.32, *p* = 0.288; Figure 2B], but overall stronger correlations in the right than in the left hemisphere [F_(1, 14)_ = 6.83, *p* = 0.020], carried mostly by the gamma and delta bands [*F*_(1, 14)_ = 5.16, *p* = 0.039; *F*_(1, 14)_ = 8.94, *p* = 0.010 respectively]. Thus, the control group presented a hemispheric division of labor consistent with that predicted by Poeppel (2003) and observed by Giraud and collaborators (Giraud et al., 2007; Morillon et al., 2010), while the dyslexic group did not.

**Table 2:** **Mean values of partial correlations**.

	**Controls (*n* = 15)**				**Dyslexics (*n* = 17)**			
	**Left**		**Right**		**Left**		**Right**	
	**Mean**	**sd**	**Mean**	**sd**	**Mean**	**sd**	**Mean**	**sd**
Gamma	0.03	0.08	0.02	0.10	−0.02	0.06	0.01	0.05
Theta	0.00	0.06	0.03	0.06	0.03	0.05	0.03^*^	0.05
Delta	0.01	0.06	0.03^*^	0.05	0.01	0.05	0.04^*^	0.06

*Averaged partial correlation over areas in left and right hemispheres of both groups*.

*Stars indicate values significantly different from 0 (p < 0.05)*.

To further interpret this triple interaction, we first compared the neural organization for each frequency band between groups by testing group × hemisphere interactions for each frequency band. For the gamma band (Figure 2C), there was a hemisphere × group interaction [*F*_(1, 28)_ = 5.30, *p* = 0.029], with a marginally significant group difference in the left [Controls > Dyslexics, *F*_(1)_ = 3.66, *p* = 0.066, effect size: *d* = 0.71] but not in the right hemisphere [*F*_(1)_ = 0.29, *p* = 0.867]. However, the theta and delta bands (Figures 2D,E) showed no significant group × hemisphere interaction [*F*_(1, 28)_ = 2.80, *p* = 0.105 and F_1, 28_ = 0.001, *p* = 0.937, respectively]. Thus, the difference between the two groups lies primarily in the lateralization of cortical responses to the gamma band.

Finally, in order to fully describe the triple interaction, we tested the group × frequency interaction, hemisphere by hemisphere. In the left hemisphere, we observed a group × frequency interaction [*F*_(1.43, 27.57)_ = 4.39, *p* = 0.030], with marginally stronger correlations for the gamma band in controls than in dyslexics as described in the paragraph above. However, in the right hemisphere we observed no group × frequency interaction [*F*_(1.52, 27.48)_ = 0.17, *p* = 0.781]. This suggests that the cortical disorganization of the auditory cortex in dyslexia affects the left more than the right hemisphere.

Because hand preference is to some extent linked with language laterality, we also investigated hand preference effects. We found an area × hemisphere × hand preference interaction [*F*_(2.20, 26.80)_ = 6.035, *p* = 0.003), reflecting over both groups stronger right than left correlations between EEG power and the BOLD signal for right-handers in area Te1.0 and Te1.2 [*F*_(1, 19)_ = 8.35, *p* = 0.009; *F*_(1, 19)_ = 13.87, *p* = 0.001, respectively], and stronger right than left correlations for left-handers in area Te1.1 and Te3 [*F*_(1, 7)_ = 5.67, *p* = 0.049; *F*_(1, 7)_ = 10.06, *p* = 0.016, respectively]. There was also a hemisphere × hand preference interaction in Te1.2 [*F*_(1, 28)_ = 9.12, *p* = 0.005], with a trend in the left hemisphere for stronger correlations in left handed than in right handed subjects [*F*_(1)_ = 3.56, *p* = 0.069].

Finally, because of the suspicion that males and females might have partly different brain bases for dyslexia (Humphreys et al., 1990; Altarelli et al., 2013; Evans et al., 2013) we checked whether there was any sex effect. No main effect of sex and no significant interaction involving this factor were observed, whether groups were considered together or apart. Thus, the results obtained seem to hold equally across males and females.

## Discussion

In a previous study (Lehongre et al., 2011) we observed that dyslexic subjects had disrupted ASSRs at phoneme-relevant rates (low gamma around 30 Hz). While controls presented a left dominance in low-gamma range oscillations, in accordance with AST theory (Poeppel, 2003), dyslexic individuals showed no asymmetry or a right dominance. However, that study relied on the processing of an unnatural sound, a white noise that was modulated in amplitude. Furthermore, that the noise was modulated from 10 to 80 Hz made it impossible for us to test brain responses in frequency bands beyond this range. In particular, we could not test Goswami (2011) hypothesis that oscillations at syllable-relevant rates (delta and theta, 1–7 Hz) were disrupted. In the current study, we used more ecological audiovisual stimuli in which sounds were essentially natural conversational speech that better reflects situation outside the laboratory, and we were able to analyze at once the whole range of frequencies that are suspected to be altered in dyslexia, in Heschl's gyrus and the *planum temporale*. By using natural speech we preferentially induce those oscillations that are prominent in speech envelope (delta/theta), and periodically reset by speech onsets. As gamma power is both evoked by speech edges and controlled by theta modulations, it also follows speech modulations in primary auditory cortex (Giraud and Poeppel, 2012).

While our results do not show a clear-cut group difference in all of the conditions examined, they do show significant interactions that, in our view, constitute stronger results than a group difference in a single hand-picked condition. Our main finding is a group × hemisphere × frequency interaction, indicating that dyslexics differ from controls in the way the processing of the three target frequency bands is distributed across the two hemispheres. While control subjects show a frequency × hemisphere interaction, with a right dominance only for theta oscillations, dyslexic subjects show the same correlation patterns across the three bands, with a global dominance of the right hemisphere, suggesting a different pattern of hemispheric specialization.

With respect to the two target hypotheses on dyslexia, we find that dyslexic individuals' auditory cortical function differs from controls more in the left than in the right auditory cortex (group × frequency interaction in the left hemisphere only), and more for the gamma than for the delta and theta bands (group × hemisphere interaction for the gamma band only). The present results hence are consistent with our previous conclusion (Lehongre et al., 2011), that dyslexia reflects a primary disruption of left-hemisphere based gamma band oscillations.

Exploring low frequency oscillations in the delta (1–3 Hz) and theta ranges (4–7 Hz), we confirmed a right dominance for delta-theta oscillations in the auditory cortex of both controls and dyslexics (Poeppel, 2003). However, as there was neither a group × hemisphere interaction for the delta and theta bands, nor any hint of a group difference in the right hemisphere, the present results do not lend much support to the hypothesis of a right-hemisphere based, delta or theta oscillation disruption (Goswami, 2011).

One possibility to explain the conflicting results between studies might be a language difference. Indeed studies reporting group differences in delta-theta frequency bands were carried out in English only (Abrams et al., 2009; Hamalainen et al., 2012). It might be that speakers of a stress-timed language like English acquire stronger sensitivity to delta and theta rhythms than those of a syllable-timed language like French. However, this hypothesis is not consistent with the results obtained in (stress-timed) Dutch speakers by Poelmans et al. (2012). Another parameter to take into account is that the BOLD signal is described as mostly driven by oscillations in the beta/gamma band (Magri et al., 2012). This might induce both greater EEG/BOLD correlations and a better signal/noise ratio for this frequency band than for the delta/theta range, and therefore increase our statistical power to detect group differences in the gamma band. However, we do not find in our data any evidence of greater correlations in the gamma than in the delta-theta bands.

Our study also differs from the other EEG/MEG studies, as we did not analyze simple EEG power spectrum. The reason is that our stimulus being conversational speech, there is no multiply-repeated stimulus that enables a well-defined spectro-temporal analyzis of the evoked response. We did not analyze the phase locking with the speech envelope either, because this would provide information about low frequencies (Howard and Poeppel, 2012), but not the gamma band, and we wanted to compare both frequency bands. Our analysis of correlations between the EEG power time course and the BOLD signal allowed us to estimate the degree to which activations in specific brain regions reflect cortical oscillations in given frequency bands. The EEG signal alone lacks spatial resolution, and the BOLD signal alone reflects much more than cortical oscillations. Here we were able to interrogate auditory cortex very precisely, as variations in EEG/BOLD correlations presumably reflect variations in cortical oscillations in the specific areas of interest, that are probably related to the fluctuations in amplitude of the speech envelope modulations.

To what extent do our results depend on the naturalistic audiovisual stimuli used here, as opposed to amplitude-modulated sounds used in previous studies? Our exclusive focus on primary and secondary auditory cortex makes it unlikely that we should observe direct responses to either the higher-level linguistic information, or the visual information. However, it is plausible that the access to semantic information and the presence of synchronized visual information (lip movements) may have served to enhance the responses to the auditory stimuli, thereby improving the signal/noise ratio.

As described by other functional and anatomical studies, many measures highlight a weak or reversed brain lateralization related to language in dyslexics, including in the *planum temporale*, labeled Te3 here (Galaburda et al., 1985, 1987; Altarelli et al., submitted). The lack of hemispheric specialization for delta, theta, and gamma oscillations could affect the efficiency of the auditory processing based on a dual-scale temporal integration (Poeppel, 2003). One possible consequence of this deficit of low gamma oscillations in dyslexics' left hemisphere is to lead them to segment the auditory input into non-standard phonemic units, hence distorting the very format of their phonological representations. This would be consistent with the general understanding of the phonological deficit in dyslexia (Mody et al., 1997; Adlard and Hazan, 1998; Serniclaes et al., 2004; Vellutino et al., 2004). Another possibility would be that such cortical oscillations do not directly affect the format of phonological representations, but their salience or their availability for downstream cognitive processes. Thus, the disruption of phoneme-relevant cortical oscillations is also compatible with the view that dyslexics' phonological representations are essentially normal, but that phonemic units are more difficult to rapidly access, to pay attention to, and to manipulate for dyslexic individuals (Ramus and Szenkovits, 2008; Ramus and Ahissar, 2012; Giraud and Ramus, 2013).

## Conclusion

In this study, we used a combined EEG-fMRI paradigm to measure the oscillations of auditory cortical areas in response to speech stimuli in dyslexic and in control adult participants, in three frequency bands of interest: gamma (25–35 Hz), theta (4–7 Hz) and delta (1–3 Hz). We first confirmed the presence of an auditory sampling impairment in dyslexia, using natural speech listening conditions. Secondly, we further confirmed that this impairment consisted mainly in a in a reduced responsiveness of left auditory cortex to gamma oscillations. On the other hand, we found little evidence for a disruption in auditory sampling in the delta and theta rates. Our results are consistent with a large literature showing the difficulties of dyslexic individuals with the representation, processing, or access to phonemic units, and provide a possible brain basis for this phenomenon.

### Conflict of interest statement

The authors declare that the research was conducted in the absence of any commercial or financial relationships that could be construed as a potential conflict of interest.

## References

[B1] Abrams D. A. Nicol T. Zecker S. Kraus N. (2008). Right-hemisphere auditory cortex is dominant for coding syllable patterns in speech. J. Neurosci. 28, 3958–3965 10.1523/JNEUROSCI.0187-08.2008 18400895 PMC2713056

[B2] Abrams D. A. Nicol T. Zecker S. Kraus N. (2009). Abnormal cortical processing of the syllable rate of speech in poor readers. J. Neurosci. 29, 7686–7693 10.1523/JNEUROSCI.5242-08.2009 19535580 PMC2763585

[B3] Adlard A. Hazan V. (1998). Speech perception in children with specific reading difficulties (dyslexia). Q. J. Exp. Psychol. A 51, 153–177 9532966 10.1080/713755750

[B5] Altarelli I. Monzalvo K. Iannuzzi S. Fluss J. Billard C. Ramus F. (2013). A functionally guided approach to the morphometry of occipito-temporal regions in developmental dyslexia: evidence for differential effects in boys and girls. J. Neurosci. 10.1523/JNEUROSCI.5854-12.2013 23825432 PMC6618608

[B6] Babiloni C. Stella G. Buffo P. Vecchio F. Onorati P. Muratori C. (2012). Cortical sources of resting state EEG rhythms are abnormal in dyslexic children. Clin. Neurophysiol. 123, 2384–2391 10.1016/j.clinph.2012.05.002 22658819

[B7] Brett M. Anton J.-L. Valabregue R. Poline J.-B. (2002). “Region of interest analysis using an SPM toolbox,” in Presented at the 8th International Conferance on Funct-ional Mapping of the Human Brain, Vol. 16 (Sendai). Available on CD-ROM in NeuroImage, abstract 497.

[B8] Darki F. Peyrard-Janvid M. Matsson H. Kere J. Klingberg T. (2012). Three dyslexia susceptibility genes, DYX1C1, DCDC2, and KIAA0319, affect temporo-parietal white matter structure. Biol. Psychiatry 72, 671–676 10.1016/j.biopsych.2012.05.008 22683091

[B9] Dunn L. M. Thériault C. M. Dunn L. M. (1993). Echelle de vocabulaire en images Peabody (EVIP). Toronto, ON: Psycan

[B10] Eckert M. (2004). Neuroanatomical markers for dyslexia: a review of dyslexia structural imaging studies. Neuroscientist 10, 362–371 10.1177/1073858404263596 15271263

[B11] Evans T. M. Flowers D. L. Napoliello E. M. Eden G. F. (2013). Sex-specific gray matter volume differences in females with developmental dyslexia. Brain Struct. Funct. [Epub ahead of print]. 10.1007/s00429-013-0552-4 23625146 PMC3775969

[B12] Frederickson N. Frith U. Reason R. (1997). Phonological Assessment Battery. Windsor: nferNelson

[B13] Galaburda A. M. Corsiglia J. Rosen G. D. Sherman G. F. (1987). Planum temporale asymmetry: reappraisal since Geschwind and Levitsky. Neuropsychologia 25, 853–868 10.1016/0028-3932(87)90091-1

[B14] Galaburda A. M. Sherman G. F. Rosen G. D. Aboitiz F. Geschwind N. (1985). Developmental dyslexia: four consecutive patients with cortical anomalies. Ann. Neurol. 18, 222–233 10.1002/ana.410180210 4037763

[B15] Giraud A. L. Kleinschmidt A. Poeppel D. Lund T. E. Frackowiak R. S. Laufs H. (2007). Endogenous cortical rhythms determine cerebral specialization for speech perception and production. Neuron 56, 1127–1134 10.1016/j.neuron.2007.09.038 18093532

[B16] Giraud A. L. Poeppel D. (2012). Cortical oscillations and speech processing: emerging computational principles and operations. Nat. Neurosci. 15, 511–517 10.1038/nn.3063 22426255 PMC4461038

[B17] Giraud A. L. Ramus F. (2013). Neurogenetics and auditory processing in developmental dyslexia. Curr. Opin. Neurobiol. 23, 37–42 10.1016/j.conb.2012.09.003 23040541

[B18] Goswami U. (2011). A temporal sampling framework for developmental dyslexia. Trends Cogn. Sci. 15, 3–10 10.1016/j.tics.2010.10.001 21093350

[B19] Goswami U. Gerson D. Astruc L. (2010). Amplitude envelope perception, phonology and prosodic sensitivity in children with developmental dyslexia. Read. Writ. 23, 995–1019 10.1007/s11145-009-9186-6

[B20] Goswami U. Wang H. L.S. Cruz A. Fosker T. Mead N. Huss M. (2011). Language-universal sensory deficits in developmental dyslexia: English, Spanish, and Chinese. J. Cogn. Neurosci. 23, 325–337 10.1162/jocn.2010.21453 20146613

[B21] Hamalainen J. A. Rupp A. Soltesz F. Szucs D. Goswami U. (2012). Reduced phase locking to slow amplitude modulation in adults with dyslexia: an MEG study. Neuroimage 59, 2952–2961 10.1016/j.neuroimage.2011.09.075 22001790

[B22] Han J. Mody M. Ahlfors S.P. (2012). Gamma phase locking modulated by phonological contrast during auditory comprehension in reading disability. Neuroreport 23, 851–856 10.1097/WNR.0b013e32835818e1 22889887 PMC4043393

[B23] Heim S. Friedman J.T. Keil A. Benasich A. A. (2011). Reduced sensory oscillatory activity during rapid auditory processing as a correlate of language-learning impairment. J. Neurolinguistics 24, 538–555 10.1016/j.jneuroling.2010.09.006 21822356 PMC3150564

[B24] Herrmann C. S. Debener S. (2008). Simultaneous recording of EEG and BOLD responses: a historical perspective. Int. J. Psychophysiol. 67, 161–168 10.1016/j.ijpsycho.2007.06.006 17719112

[B25] Howard M. F. Poeppel D. (2012). The neuromagnetic response to spoken sentences: co-modulation of theta band amplitude and phase. Neuroimage 60, 2118–2127 10.1016/j.neuroimage.2012.02.028 22374481 PMC3593735

[B26] Humphreys P. Kaufmann W. E. Galaburda A. M. (1990). Developmental dyslexia in women: neuropathological findings in three patients. Ann. Neurol. 28, 727–738 10.1002/ana.410280602 2285260

[B27] Laufs H. (2012). A personalized history of EEG-fMRI integration. Neuroimage 62, 1056–1067 10.1016/j.neuroimage.2012.01.039 22266176

[B28] Laufs H. Krakow K. Sterzer P. Eger E. Beyerle A. Salek-Haddadi A. (2003). Electroencephalographic signatures of attentional and cognitive default modes in spontaneous brain activity fluctuations at rest. Proc. Natl. Acad. Sci. U.S.A. 100, 11053–11058 10.1073/pnas.1831638100 12958209 PMC196925

[B29] Lee J. H. Durand R. Gradinaru V. Zhang F. Goshen I. Kim D. S. (2010). Global and local fMRI signals driven by neurons defined optogenetically by type and wiring. Nature 465, 788–792 10.1038/nature09108 20473285 PMC3177305

[B30] Lefavrais P. (1967). Test de l'Alouette, 2nd Edn. Paris: Editions du Centre de Psychologie Appliquée

[B31] Lehongre K. Ramus F. Villiermet N. Schwartz D. Giraud A. L. (2011). Altered low-gamma sampling in auditory cortex accounts for the three main facets of dyslexia. Neuron 72, 1080–1090 10.1016/j.neuron.2011.11.002 22196341

[B32] Lindgren S. D. De Renzi E. Richman L. C. (1985). Cross-national comparisons of developmental dyslexia in Italy and the United States. Child Dev. 56, 1404–1417 10.2307/1130460 3878269

[B33] Linkersdorfer J. Lonnemann J. Lindberg S. Hasselhorn M. Fiebach C. J. (2012). Grey matter alterations co-localize with functional abnormalities in developmental dyslexia: an ALE meta-analysis. PLoS ONE 7:e43122 10.1371/journal.pone.0043122 22916214 PMC3423424

[B34] Logothetis N. K. (2010). Bold claims for optogenetics. Nature 468, E3–4; discussion: E4–E5. 21107378 10.1038/nature09532

[B35] Magri C. Schridde U. Murayama Y. Panzeri S. Logothetis N. K. (2012). The amplitude and timing of the BOLD signal reflects the relationship between local field potential power at different frequencies. J. Neurosci. 32, 1395–1407 10.1523/JNEUROSCI.3985-11.2012 22279224 PMC6796252

[B36] Mandelkow H. Halder P. Boesiger P. Brandeis D. (2006). Synchronization facilitates removal of MRI artefacts from concurrent EEG recordings and increases usable bandwidth. Neuroimage 32, 1120–1126 10.1016/j.neuroimage.2006.04.231 16861010

[B37] Mody M. Studdert-Kennedy M. Brady S. (1997). Speech perception deficits in poor readers: auditory processing or phonological coding? J. Exp. Child Psychol. 64, 199–231 10.1006/jecp.1996.2343 9120381

[B38] Morillon B. Lehongre K. Frackowiak R. S. J. Ducorps A. Kleinschmidt A. Poeppel D. (2010). Neurophysiological origin of human brain asymmetry for speech and language. Proc. Natl. Acad. Sci. U.S.A. 107, 18688–18693 10.1073/pnas.1007189107 20956297 PMC2972980

[B39] Oostenveld R. Fries P. Maris E. Schoffelen J. M. (2011). FieldTrip: open source software for advanced analysis of MEG, EEG, and invasive electrophysiological data. Comput. Intell. Neurosci. 2011, 156869 10.1155/2011/156869 21253357 PMC3021840

[B40] Pennington B. F. (2006). From single to multiple deficit models of developmental disorders. Cognition 101, 385–413 10.1016/j.cognition.2006.04.008 16844106

[B41] Pinel P. Fauchereau F. Moreno A. Barbot A. Lathrop M. Zelenika D. (2012). Genetic variants of FOXP2 and KIAA0319/TTRAP/THEM2 locus are associated with altered brain activation in distinct language-related regions. J. Neurosci. 32, 817–825 10.1523/JNEUROSCI.5996-10.2012 22262880 PMC6621137

[B42] Poelmans H. Luts H. Vandermosten M. Boets B. Ghesquiere P. Wouters J. (2012). Auditory steady state cortical responses indicate deviant phonemic-rate processing in adults with dyslexia. Ear Hear. 33, 134–143 10.1097/AUD.0b013e31822c26b9 21844810

[B43] Poeppel D. (2003). The analysis of speech in different temporal integration windows: cerebral lateralization as “asymmetric sampling in time.” Speech Commun. 41, 245–255 10.1016/S0167-6393(02)00107-3

[B44] Ramus F. Ahissar M. (2012). Developmental dyslexia: the difficulties of interpreting poor performance, and the importance of normal performance. Cogn. Neuropsychol. 29, 104–122 10.1080/02643294.2012.677420 22559749

[B45] Ramus F. Szenkovits G. (2008). What phonological deficit? Q. J. Exp. Psychol. 61, 129–141 10.1080/17470210701508822 18038344

[B46] Raven J. Raven J. C. Court J. H. (1998). Advanced Progressive Matrices. Oxford: Oxford Psychologists Press

[B47] Richardson F. M. Price C. J. (2009). Structural MRI studies of language function in the undamaged brain. Brain Struct. Funct. 213, 511–523 10.1007/s00429-009-0211-y 19618210 PMC2749930

[B48] Richlan F. Kronbichler M. Wimmer H. (2011). Meta-analyzing brain dysfunctions in dyslexic children and adults. Neuroimage 56, 1735–1742 10.1016/j.neuroimage.2011.02.040 21338695

[B49] Rosa M. J. Daunizeau J. Friston K. J. (2010a). EEG-fMRI integration: a critical review of biophysical modeling and data analysis approaches. J. Integr. Neurosci. 9, 453–476 10.1142/S0219635210002512 21213414

[B50] Rosa M. J. Kilner J. Blankenburg F. Josephs O. Penny W. (2010b). Estimating the transfer function from neuronal activity to BOLD using simultaneous EEG-fMRI. Neuroimage 49, 1496–1509 10.1016/j.neuroimage.2009.09.011 19778619 PMC2793371

[B51] Serniclaes W. Van Heghe S. Mousty P. Carre R. Sprenger-Charolles L. (2004). Allophonic mode of speech perception in dyslexia. J. Exp. Child Psychol. 87, 336–361 10.1016/j.jecp.2004.02.001 15050458

[B52] Soroli E. Szenkovits G. Ramus F. (2010). Exploring dyslexics' phonological deficit III: foreign speech perception and production. Dyslexia 16, 318–340 10.1002/dys.415 20957686

[B53] Tallal P. (1980). Auditory temporal perception, phonics, and reading disabilities in children. Brain Lang. 9, 182–198 10.1016/0093-934X(80)90139-X 7363063

[B54] Telkemeyer S. Rossi S. Koch S. P. Nierhaus T. Steinbrink J. Poeppel D. (2009). Sensitivity of newborn auditory cortex to the temporal structure of sounds. J. Neurosci. 29, 14726–14733 10.1523/JNEUROSCI.1246-09.2009 19940167 PMC6666009

[B55] Vellutino F. R. Fletcher J. M. Snowling M. J. Scanlon D. M. (2004). Specific reading disability (dyslexia): what have we learned in the past four decades? J. Child Psychol. Psychiatry 45, 2–40 10.1046/j.0021-9630.2003.00305.x 14959801

[B56] Wagner R. K. Torgesen J. K. (1987). The nature of phonological processing and its causal role in the acquisition of reading skills. Psychol. Bull. 101, 192–212 10.1037/0033-2909.101.2.19222209402

[B57] Wechsler D. (2000). WAIS-III: Echelle de l'intelligence de Wechsler pour adultes. Troisième Edn. Paris: Editions du Centre de Psychologie Appliquée

[B58] World Health Organization. (2011). International Statistical Classification of Diseases and Related Health Problems—10th revision. Geneva: World Health Organization

